# Pain control after total knee arthroplasty: a randomized trial comparing local infiltration anesthesia and continuous femoral block

**DOI:** 10.3109/17453674.2011.581264

**Published:** 2011-09-02

**Authors:** Fatin Affas, Eva-Britt Nygårds, Carl-Olav Stiller, Per Wretenberg, Christina Olofsson

**Affiliations:** ^1^Department of Anesthesiology and Intensive Care; ^2^Department of Medicine, Clinical Pharmacology Unit; ^3^Department of Molecular Medicine, Section of Orthopaedics, Karolinska Institute and Karolinska University Hospital, Solna, Sweden; Correspondence: per.wretenberg@karolinska.se

## Abstract

**Background and purpose:**

Pain after total knee arthroplasty (TKA) is usually severe, and epidural analgesia or femoral nerve block has been considered to be an effective pain treatment. Recently, local infiltration analgesia (LIA) has become increasingly popular but the outcome of this method regarding the analgesic effect has not been fully evaluated. We compared local infiltration analgesia and femoral block with regard to analgesia and morphine demand during the first 24 h after TKA.

**Methods:**

40 patients undergoing TKA under spinal anesthesia were randomized to receive femoral nerve block (group F) or peri- and intraarticular infiltration analgesia (group LIA) with a mixture containing ropivacaine, ketorolac, and epinephrine. All patients had access to intravenous patient-controlled analgesia (PCA) with morphine postoperatively. Pain intensity at rest and upon movement was assessed on a numeric rating scale (0–10) on an hourly basis over 24 h if the patients were awake.

**Results:**

The average pain at rest was marginally lower with LIA (1.6) than with femoral block (2.2). Total morphine consumption per kg was similar between the 2 groups. Ancillary analysis revealed that 1 of 20 patients in the LIA group reported a pain intensity of > 7 upon movement, as compared to 7 out of 19 in the femoral block group (p = 0.04).

**Interpretation:**

Both LIA and femoral block provide good analgesia after TKA. LIA may be considered to be superior to femoral block since it is cheaper and easier to perform.

Pain after total knee arthroplasty (TKA) is usually severe and difficult to manage, and insufficient pain relief may delay recovery. The most effective pain treatment has traditionally been epidural analgesia or femoral nerve block (Singelyn et al. 1998, Ganapathy et al. 1999, Chelly et al. 2001, Davies et al. 2004, Ilfeld et al. 2006) in combination with opioid analgesics and non-steroidal anti-inflammatory drugs (NSAIDs, cyclooxygenase (cox) inhibitors). Each of these methods has its specific side effects. Urinary retention and muscular weakness are often reported after epidural analgesia. Unpleasant numbness of a large part of the lower extremity is common after femoral block. Opioid analgesics often cause sedation, nausea and vomiting, and also urinary retention. Non-selective cox inhibitors may cause gastrointestinal bleeding, renal complications, and epidural hematoma, especially in combination with anti-thrombotic prophylaxis with low-molecular-weight heparin (Afzal et al. 2006).

An alternative method for postoperative pain relief after TKA, which has attracted growing interest in recent years, is multimodal wound infiltration analgesic technique consisting of peri- and intraarticular infiltration of local anesthetics and NSAID in the knee (LIA) (Andersen et al. 2008a, b, Kerr and Kohan 2008). This technique appears to offer several advantages over traditional methods, since the analgesia affects only the surgical area with limited interference of the muscle strength. Thus, easier rehabilitation of the operated extremity and earlier discharge from the hospital can be expected (Reilly et al. 2005, Essving et al. 2009). Furthermore, recent studies have shown that the LIA technique reduces the requirement for postoperative analgesia with opioids (Tanaka et al. 2001, Busch et al. 2006, Vendittoli et al. 2006).

Only a few investigators have randomly compared LIA with other methods with proven analgesic effect, such as femoral block or epidural analgesia (Parvataneni et al. 2007, Toftdahl et al 2007). Parvatanemi and collaborators (2007) have shown that a combination of a femoral block and local administration of bupivacaine, morphine, and epinephrine results in better pain relief and patient satisfaction than femoral block. Toftdahl and collaborators (2007) presented data suggesting that LIA with ropivacaine, ketorolac, and epinephrine results in faster postoperative activation, as indicated by being better able to walk more than 3 m on the first postoperative day as compared to femoral block. A retrospective comparison (DeWeese et al. 2001) indicated that epidural anesthesia with fentanyl and bupivacaine resulted in better pain relief and less use of other analgesics than did continuous infiltration of the knee with bupivacaine.

Femoral block is known to be an effective pain treatment after TKA (Szczukowski et al. 2004, Navas et al. 2005, Duarte et al. 2006). We compared the LIA technique with femoral block regarding efficacy of pain management at rest and upon movement after TKA. We also investigated whether LIA reduced the demand for intravenous morphine, administered via a patient-controlled analgesia (PCA) pump during the first 24 h postoperatively.

## Patients

This randomized parallel clinical 1:1 trial was used to compare two protocols of postoperative pain relief after total knee arthroplasty. The inclusion criteria were as follows: patients with osteoarthritis or rheumatoid arthritis scheduled for primary unilateral elective total knee arthroplasty under spinal anesthesia, American Society of Anaesthesiologists (ASA) classification I–III, and more than 18 years old. Exclusion criteria were allergy or intolerance to one of the study drugs, renal insufficiency, epilepsy, language difficulty, mental illness, dementia, QT interval on ECG > 450 msec before start. After giving oral and written informed consent, 40 patients scheduled for primary unilateral total knee arthroplasty were randomly assigned to 2 groups of postoperative pain management immediately before the surgical procedure.

The setting of this single-site academic trial was the orthopedics clinic at Karolinska University Hospital in Solna. The first patient was included on January 15, 2007 and the final patient was included on March 25, 2008.

### Randomization

The randomization sequence was determined by mixing 40 tickets, 20 labeled “F” and 20 labeled “LIA” in sealed opaque envelopes, and drawing one envelope at a time. The anesthesiologist performing the spinal anesthesia and femoral block or supervising the LIA technique did not participate in the randomization procedure.

### Interventions

Group F received a femoral block with ropivacaine (Narop; Astra Zeneca) and group LIA received peri- and intraarticular infiltration with ropivacaine + ketorolac (Toradol; Roche) and epinephrine (Adrenalin; NM Pharma).

### Preparation

Before induction of spinal anesthesia, monitoring of oxygen saturation, blood pressure, and electrocardiogram (ECG) was started. Sedation was induced with midazolam (Midazolam; Alpharma), 1–2 mg intravenously. The level of spinal anesthesia was L2-L3 or L3-L4. Isobaric bupivacaine (Marcain Spinal; Astra Zeneca), 5 mg/mL at a volume of 3 mL, was injected with the patient lying with the operating side upwards.

Before the start of the operation, all patients were sedated with midazolam or propofol (Propofol; Abbot), maintaining spontaneous ventilation. All patients received 2 g dicloxacillin intravenously before surgery. Antithrombotic therapy with low-molecular-weight heparin (LMWH), enoxaparinnatrium (Klexane; Aventis Pharma) 40 mg, started the day before surgery and was given for at least 5 days. The TKA procedure was performed following application of a thigh tourniquet, which was inflated just before skin incision and released after wound closure.

### Group F

These patients received a femoral nerve block directly after spinal anesthesia. They were placed in the supine position. Under sterile conditions, the pulse of the femoral artery was identified, the needle (Plexolong Nanolin cannula facette 19G × 50 mm; Pajunk) connected to a nerve stimulator (Simplex B, serial no 17002; Braun) set up to deliver 1.2 mA was inserted cephalad, 45 degrees to skin and at the level of femoral crease, 1–1.5 cm lateral to the femoral artery pulse (Winnie et al. 1973). The femoral nerve was identified by eliciting quadriceps muscle contractions (“dancing patella”). The current was gradually reduced to achieve twitches of the quadriceps muscle at 0.2–0.4 mA and the catheter (StimuLong Sono; Pajunk) was advanced through the needle.

The connection of the nerve stimulator was changed from needle to catheter, and stimulation intensity was started at 1.2 mA until the desired motor response was obtained. Thereafter, the intensity was reduced to 0.2–0.4 mA. The catheter was secured in place with transparent dressing. After negative blood aspiration, 30 mL ropivacaine (2 mg/mL) was injected followed by 15 mL of the same concentration every 4 hours for 24 h (total dose 240 mg/24 h). All patients had a urinary bladder catheter, inserted after spinal anesthesia and removed on the day after surgery. Group F received ketorolac (10 mg intravenously) in the post-anesthetic care unit, and again after 8 h and 16 h. The total dose of ketorolac was 30 mg.

### Group LIA

These patients received peri- and intraarticular infiltration of a solution containing 150 mL ropivacaine (2 mg/mL), 1 ml ketorolac (30 mg/mL), and 5 ml epinephrine (0.1 mg/mL). This solution was prepared by the operation nurse before the start of the surgical procedure. The solution was given sequentially: 30 mL was injected intracutaneously at the start of the operation, 80 mL was injected into the posterior part of the capsule, close to the incision line, in the vastus intermedius and lateralis and around the collateral ligaments before cementation, and 46 mL was instilled through an intraarticular catheter (epidural catheter gauge 16) inserted at the end of the surgical procedure. The total dose of ropivacaine during the first 24 h postoperatively was 300 mg and the total dose of ketorolac was 30 mg.

### Recovery

In the recovery room, all patients were provided with a patient-controlled analgesia (PCA) morphine pump (Abott Pain Manager; Abbot Laboratories) programmed to give an intravenous bolus of morphine (2 mg/dose) on demand with a lock-out time of 6 min and maximum dose of 35 mg over 4 h. All patients were introduced to the PCA technique and encouraged to use it as often as needed. After 24 hours, PCA pump use was verified with a printout of all doses of morphine and their time of administration. In addition to PCA, all patients received paracetamol (1 g × 4), either orally or intravenously.

All patients were informed preoperatively by the nurse about pain assessment using a numeric rating scale (NRS): 0 = no pain and 10 = worst imaginable pain, based on the visual analog scale. NRS score at rest or upon movement during the first 24 h was recorded on an hourly basis by the patients, if awake.

ECG was performed preoperatively, 2 h after the end of surgery in the recovery room and 24 h postoperatively in the ward. All patients received postoperative physiotherapy, which started on the morning after operation.

### Statistics

Power analysis was performed using average VAS/NRS score during 24 h as the primary variable. A previous study of patients undergoing knee arthroplasty treated with a femoral nerve block reported a mean visual analog scale score of 3.6 (SD 1.1) upon movement, at 24 h (Singelyn et al. 1998). We wanted to be able to detect a difference of 1 unit between LIA and femoral block. A sample size of 20 in each group would have 80% power to detect a difference between means of 1.0 with a significance level (alpha) of 0.05 (two-tailed) using the unpaired Student's t-test (GraphPad StatMate 1.0; GraphPad, San Diego, CA).

### Outcomes

Primary outcome. Differences in average pain intensity at rest and upon movement during the first 24 h after TKA were analyzed with the Mann-Whitney U-test. As we had 2 primary efficacy outcomes, a significance level of p < 0.02 was chosen for each analysis. No data were imputed for the primary outcome if the patient was asleep or unable to record NRS.

Secondary outcome. Total morphine use via the intravenous PCA pump during the 24-h period in the 2 study arms were analyzed with the Mann-Whitney U-test. Descriptive statistics were used to describe the morphine dose per kg in each study arm.

### Ancillary analyses

These were as follows. 1. Average pain intensity in 24 hours following imputation of missing data. Missing data was replaced by “0” if the patient was asleep. The most recent NRS score obtained was used to replace missing if pain rating was not provided due to other reasons. 2. The fraction of patients who reported a degree of pain intensity of < 5 at rest and during movement, i.e. mild pain intensity (Jensen et al. 2003) in the 2 study groups was compared with Fisher's exact test. 3. The fraction of patients who reported a pain intensity of > 7 at rest and during movement, i.e. severe pain (Jensen et al. 2003) in the two study groups was compared with Fisher's exact test.

### Safety monitoring

We monitored indications of cardiac arrhythmias and incidence of reported adverse events.

### Ethics

This trial (protocol no. 4773 KCR S2006-011) was conducted according to the Helsinki declaration and was approved by the regional ethics committee of the Karolinska Institute (EPN 151:2006/34610) and the Swedish Medical Product Agency (EudraCT 2006-002581-19). The trial was monitored by the Karolinska Trial Alliance. This trial was not registered in the FDA database of clinical trials.

## Results

40 patients participated in this trial, which was conducted. 20 patients were randomized to LIA and 20 to femoral block. All patients completed the study. Data were analyzed according to strict intention-to-treat (sITT) analysis according to Herman et al. (2009). One patient in the F group had a history of insensitivity to pain. He did not demand any morphine by PCA and pain was assessed as 0 on the NRS at all time points. The most recent NRS score obtained was used to replace missing if pain rating was not provided due to other reasons The most recent NRS score obtained was used to replace missing if pain rating was not provided due to other reasons and there was no use of morphine by PCA and 0 on the NRS at all times. This rare condition was not detected by the screening physician at inclusion in the trial. Exclusion of the data obtained from this patient and analysis according to modified intention-to-treat method (mITT) (Herman et al. (2009) did not affect the outcome data (data not shown). The baseline characteristics and the demographic data of the patients were similar in both groups—except for the average weight, which was higher in the femoral block group (Table 1). On average, 6 hourly time points of 24 were missing due to the patients being asleep or unable to fill in the CRF due to activities outside the ward.

**Table 1.** Baseline patient demographics

	Femoral block	LIA
Sex (M/F)	8/12	11/9
Age, mean (range)	69 (53–88)	67 (29–85)
Weight in kg, mean (SD)	83 (13)	78 (18)
Length in cm, mean (SD)	168 (9)	171 (11)
BMI (mean)	27	27
ASA I / II / III	2 / 8 / 10	3 / 7 / 10
RA / OA	3 / 17	6 / 14

M: male; F: female; RA: rheumatoid arthritis; OA: osteoarthritis; ASA: American Society of Anaesthesiologists classification.

### Primary outcome

**Figure 1.**
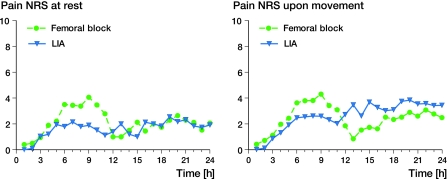
Average pain score (NRS) at rest and upon movement over 24 h after surgery. If the patient was sleeping, no data were recorded (n = 20 in each group).

The average degree of pain intensity during the first postoperative day was low in both groups. The average degree of pain intensity at rest and upon movement was similar (Table 2 and Figure 1).

**Table 2.** Primary outcome

	Femoral block n = 20	LIA n = 20
Average pain NRS at rest **^a^**	2.1 (1.4–2.9)	1.6 (1.0–2.3)
Average pain NRS upon movement **^a^**	2.4 (1.5–3.2)	2.4 (1.7–3.0)

^a^Reported data only. Missing NRS registrations due to the patients being asleep or unable to fill in the CRF due to other activities were not imputed. Data are expressed as mean (95% CI).

### Secondary outcomes

**Figure 2.**
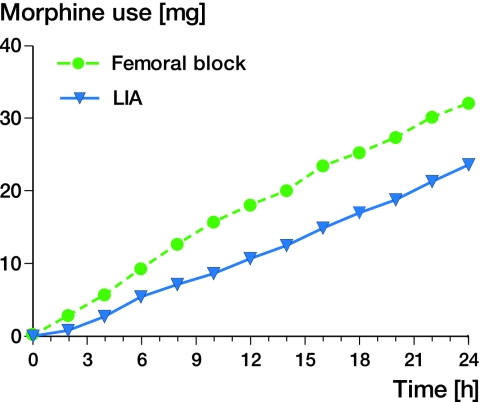
Average morphine use (PCA) in mg for the 2 groups during the first 24 h after surgery (n = 20 in each group).

The average total morphine use via the intravenous PCA pump during the first postoperative day was 10 mg higher in the femoral block group than in the LIA group, but this difference was not statistically significant (Figure 2). However, the morphine dose per kg was almost identical in both groups (Table 3).

**Table 3.** Secondary outcome

	Femoral block	LIA
Total morphine (mg)	32 (23–41)	24 (16–31)
Total morphine (mg/kg)	0.4 (0.3–0.5)	0.3 (0.2–0.4)

Total morphine refers to intravenous morphine administered via a PCA in 24 h. Data are expressed as mean (95% CI).

### Ancillary analysis

1. Imputation of missing data by “0” if the patients was asleep, and using the most recent NRS score to replae missing data if pain rating was not provided due to other reasons resulted in a mean NRS at rest of 2.2 (95% CI: 1.4–3.1) in the femoral block group and of 1.5 (CI: 0.8–2.1) in the LIA group. The average pain score upon movement, following imputation of missing observations, was 2.2 (CI: 1.5–3.0) in the femoral block group and 2.1 (CI: 1.5–2.8) in the LIA group. 2. Incidence of NRS less than 5 throughout the 24-h observation period (probably acceptable pain relief) at rest and upon movement was twice as high in the LIA group than in the femoral block group (Table 4). 3. The incidence of NRS pain intensity greater than 7 at rest on one or more occasions during the 24-h observation period was 5 times higher in the femoral block group than in the LIA group (Table 4). NRS greater than 7 upon movement was only reported by 1 of 20 patients in the LIA group and 7 of 19 patients in the femoral block group (p = 0.04, Fisher's exact test).

**Table 4.** Ancillary analysis

	Femoral block (n = 19)	LIA (n = 20)
NRS < 5 at rest	7	12
NRS < 5 upon movement	5	8
NRS > 7 at rest	5	1
NRS > 7 upon movement	7	1 **^a^**

NRS < 5 refers to the number of patients who reported a NRS lower than 5 throughout the 24 h of observation. NRS > 7 refers to the number of patients who reported pain intensity greater than 7 once or more often during the 24 h of observation.

^a^p< 0.05 (Fisher's exact test).

### Safety analysis

None of the patients had prolonged QT interval at the ECG 2 hours and 24 hours postoperatively. No adverse events were reported during the 24-h study period.

## Discussion

Our data indicate that the 2 analgesic regimens gave similar quality of pain relief during the first 24 h. Some studies have compared LIA with other pain treatments after TKA, such as systemic analgesia and placebo, and have reported superior outcome with LIA regarding postoperative analgesia and opioid consumption. The positive results in those studies are not surprising, since the analgesic treatment in the control groups mainly consisted of less effective methods of postoperative pain treatment (Lombardi et al. 2004, Vendittoli et al. 2006, Parvataneni et al. 2007, Rostlund and Kehlet 2007, Andersen et al. 2008, Kerr and Kohan 2008, Essving et al. 2009).

Both femoral block and LIA resulted in low average pain intensity during the first postoperative day. The average degree of pain intensity at rest, but not upon movement, was slightly lower in the LIA group. This small difference is probably without clinical significance. The access to PCA-administered morphine until pain was acceptably low, NRS < 4, would be expected to result in a similar degree of pain relief. The total use of morphine per kg was similar in both groups, which indicates that the pain relief of both methods was comparable. Using ancillary analysis, we found that a pain intensity of greater than 7 on NRS with movement was less common in the LIA group, a difference that reached statistical significance. This result must be regarded as hypothesis generating, and should be confirmed by a separate study.

We compared 2 routinely used methods that are both effective for postoperative pain relief in TKA. None of the patients in this study were given an analgesic other than intravenous morphine, administered through a PCA pump, and paracetamol (4 g in 24 h).

Femoral block and LIA after TKA have been compared previously (Parvataneni et al. 2007, Toftdahl et al 2007). However, in the study by Toftdahl et al., the femoral block group also received intraarticular injection of morphine (4 mg) and bupivacain (50 mg), and in the study by Parvateneni et al. a variable dose of morphine (4–10 mg) and methylprednisolone (40 mg) was given to the LIA group. In both trials, reduced opioid consumption was found with LIA. Pain relief at rest was good, but was similar in the 2 groups during the first 24 h. In accordance with our findings (with fewer patients reporting high-intensity pain on movement in the LIA group), a previous study has found better pain relief during physiotherapy after LIA (Toftdahl et al 2007).

The tendency of lower efficacy with femoral block may be due to the fact that the posterior part of the knee is innervated by the sciatic nerve. Thus, a femoral block does not cover this area and supplementary treatment with systemic analgesics such as opioids and NSAIDs is needed. In our study, all the patients in the femoral group received ketorolac in a total dose of 30 mg intravenously during the first 24 h—the same amount as administered locally in the LIA group.

One explanation as to why LIA is so effective might be that there is evidence for a clinically relevant peripheral analgesic action of intraarticular NSAIDs (Romsing et al. 2000). The analgesic effect of NSAIDs may be better after intraarticular administration than after systemic administration (Day et al. 1999). Furthermore, in a study comparing the analgesic effect of NSAIDs after wound infiltration with that after systemic administration, the result was in favor of local infiltration (Ben-David et al. 1995). Our study groups received NSAID either intravenously (group F) or via peri- and intraarticular infiltration (group LIA), but the study design was not set up to answer the question of the most effective route of administration of NSAID. Furthermore, a clear relationship between the dose of NSAIDs and their analgesic effect has been established (Collins et al. 1998). Thus, the systemic dose of NSAIDs used may have been too low for full analgesic effect in the femoral group.

The high quality of the femoral blocks in this study might be one explanation for the small difference in analgesic efficacy between the two methods. It is well known that the quality of a peripheral blockade might depend on the experience of the anesthesiologist, and all but 2 blocks were performed by an experienced anaesthesiologist (FA).

Experience is also of importance for perioperative local infiltration. When we introduced the LIA method at our institution, we noticed that surgeons also have a learning curve in doing effective local infiltration. The more experienced the surgeon is, the more effective is the postoperative pain relief. This may also be due to less tissue trauma being produced by experienced surgeons.

### Limitations of the study

Open labeled studies have a disadvantage compared to blinded studies, due the interference of expectancy of the patient and the staff in interpretation of the outcome. The patients in group F received an initial femoral nerve block followed by bolus doses of ropivacaine every 4 h during the first 24 h, and not continuous infusion of local anesthetic, which might have been more effective due to a steady concentration of ropivacaine over time and probably better pain control. The randomization procedure was simple and did not use block design, which could be a disadvantage. Furthermore, stratification according to to sex and osteoarthritis or rheumatoid arthritis might have resulted in 2 groups with less difference.

Since the number of patients with rheumatoid arthritis was higher in the LIA group, we cannot rule out that LIA or femoral block may be more effective in this patient category. The observation period of 24 h may be too short, especially with regard to adverse events. Furthermore, it would have been good to investigate whether there were differences regarding ease of rehabilitation or physical therapy.

In summary, in this randomized study we could not confirm that there was any clear superiority of perioperative infiltration of local anaesthetic (LIA) over femoral block in combination with i.v. ketorolac in total knee arthroplasty, since the two analgesic regimens gave similar quality of pain treatment during the first 24 h. However, LIA may be considered the preferred option since it is cheaper and easier to perform than femoral block. In addition, LIA involves the surgeon in alleviating postoperative pain.
